# Essential Health and Nutrition Service Provision during the COVID-19 Pandemic: Lessons from Select Ethiopian *Woredas*

**DOI:** 10.1093/cdn/nzab024

**Published:** 2021-03-18

**Authors:** Abdulhalik Workicho, Meghan J Kershaw, Lioul Berhanu, Mebit Kebede, Eileen Kennedy

**Affiliations:** Tufts University, Friedman School of Nutrition Science and Policy, Boston, MA, USA; Tufts University, Friedman School of Nutrition Science and Policy, Boston, MA, USA; Save the Children International, Addis Ababa, Ethiopia; Save the Children International, Addis Ababa, Ethiopia; Tufts University, Friedman School of Nutrition Science and Policy, Boston, MA, USA

**Keywords:** COVID-19, health systems, maternal and child health, health service delivery, nutrition services, Ethiopia

## Abstract

The world has faced a public health emergency due to the emergence of the COVID-19 pandemic. A cross-sectional study with mixed methods was conducted to review the status of maternal and child health care and nutrition service delivery during the early months of the pandemic in *woredas* (districts) targeted by the Growth through Nutrition Activity, a multi-sectoral nutrition project in Ethiopia. Comparison with the previous year showed some decline in key maternal and child health and nutrition services, with more pronounced effects during the early months of March and April before coordinated effort and standard guidance were well established. A recovery of most services was likely due in no small part to a range of mitigation interventions implemented by respective health workers and institutions, supervising government organizations, and through support from non-governmental organizations.

## Teaser

Findings from a cross-sectional, mixed-method study reviewing status of maternal and child health and nutrition service delivery in select areas of Ethiopia during the COVID-19 pandemic.

## Introduction

The world has faced an unprecedented crisis as a result of the COVID-19 pandemic, the full impact of which is only beginning to be understood (1). In the past year, national governments have adopted a variety of response actions including aggressive measures aimed at containing new infections and halting spread of the coronavirus (2). These critical efforts to mitigate the pandemic have resulted in profound effects globally, but most significantly in low- and middle-income countries. Ethiopia confirmed its first COVID-19 case in March 2020. The government declared a state of emergency on 8 April, 2020 with measures to prevent transmission including travel restrictions, border closure, suspension of public gatherings, closure of schools and universities, and requirements for social distancing. The effects of these mitigation strategies on the country's health sector were expected to be significant but remain unclear (3–8).

The Feed the Future Ethiopia Growth through Nutrition Activity, funded by the US Agency for International Development, is a multi-sectoral project aimed at improving the nutritional status of women, children, and adolescents in Ethiopia. Working in 4 key regions, Growth through Nutrition has been supporting the health system primarily through enhancing the coverage and quality of maternal and child health services, focusing on quality improvements in priority health and nutrition services at Primary Health Care Units (PHCUs) comprised of district-level health centers (HCs) and smaller local-level health posts (HPs).

Given the likely challenges to the health system due to the burden of the disease and the government-mandated mitigation strategies, it was anticipated that the provision of health and nutrition services would be challenged by staff redeployment to provide COVID-19 relief, closures of health facilities or services, and supply-chain difficulties limiting provision of care, as well as reductions in outpatient care attendance due to fear, lockdowns, and financial difficulties (9). In recognition of the burden of the pandemic on the health sector, in April 2020, the Ethiopian Federal Ministry of Health (FMoH) developed national guidelines for managing COVID-19. The national guidelines set standards for surveillance, tracing protocols, COVID treatment centers, as well as HC preparedness, community engagement, and maintaining essential services during the pandemic (10).

This study reviewed data from PHCUs on the provision of maternal and child health service delivery during the first 5 months of the COVID-19 pandemic in Ethiopian *woredas* (districts) targeted by the Growth through Nutrition Activity in order to better understand changes in service provision and areas for improved response and mitigation.

## Methods

The study was conducted in 4 regions of Ethiopia: Tigray, Amhara, Oromia, and Southern Nations, Nationalities, and Peoples’ Region. Four woredas in each of the 4 regions were purposively selected. The criteria for woreda selection included: support from Growth through Nutrition, reported cases of COVID-19, and no other recent, major natural or man-made shocks that had significantly disrupted routine health services. A total of 16 rural PHCUs were selected (1 per woreda), which included 16 HCs and 16 HPs. However, only 15 HCs were included in the final sample as 1 center had temporarily closed due to staff infection with COVID-19.

The study applied a cross-sectional study design including both quantitative and qualitative data collection methods. Experienced and trained data collectors collected quantitative data on the performance of key indicators from March to July of 2019 and 2020, retrieved from the selected health facilities records via interviews with staff, verified against documented copies. For the HC staff, interviewees included health center head or the quality improvement focal person, and for the HPs, health extension workers (HEWs). Additionally, 8 individuals were selected to participate in qualitative key informant (KI) interviews, 2 individuals from health facilities in each region. The persons selected for interviews were those expected to be the most knowledgeable about provision of health services in their respective facilities. Respondents had on average 3 years of experience in their current position, 5 had a bachelor's degree in nursing or public health, 3 were diploma nurses. Four of the respondents were female.

Due to COVID-19 precautions, interviews and data collection were done remotely over the phone in August 2020. The study received ethical review by the Tufts University Institutional Review Board and letters of support from the respective Regional Health Bureaus.

## Results

Based on data collected from PHCUs, almost all representatives reported that service delivery and utilization of health/nutrition services had been affected by the COVID-19 pandemic (14 HCs). Although not many COVID-19 cases were reported, the fear of infection among service providers was the chief reason given for disruption of service delivery/utilization in all 15 HCs. Respondents noted that panic about COVID-19 within communities was exacerbated by constant news and media attention as well as circulating rumors and misinformation, which collectively negatively influenced the perception of both service providers and clients.

This is captured in the following quotes:

… at the beginning of the pandemic there was an influence of COVID-19 on all our services. We panicked with the different news and almost stopped the service delivery especially for maternal health services. (HEW from Amhara)

The community is afraid of coronavirus infection, assuming that a patient who goes to the health center at this time would probably be infected by coronavirus. (KI from HC in Tigray)

Other reasons cited for decreased service utilization in the woredas included: shortage of PPE (9 HCs), shortage of medical supplies (7 HCs), and travel restrictions (7 HCs). The 3 main reported mitigation strategies used to combat COVID-19 included awareness creation among the staff and community (15 HCs), aggressive implementation of infection prevention (13 HCs), and increased use of PPE (9 HCs). While health workers reported being initially overwhelmed at the beginning of the pandemic, the majority (13 HCs) felt that mitigation strategies had been successful. Perceived challenges that remain are continued shortages of PPE and other medical supplies, and staff and community perception of risk.

**FIGURE 1:**
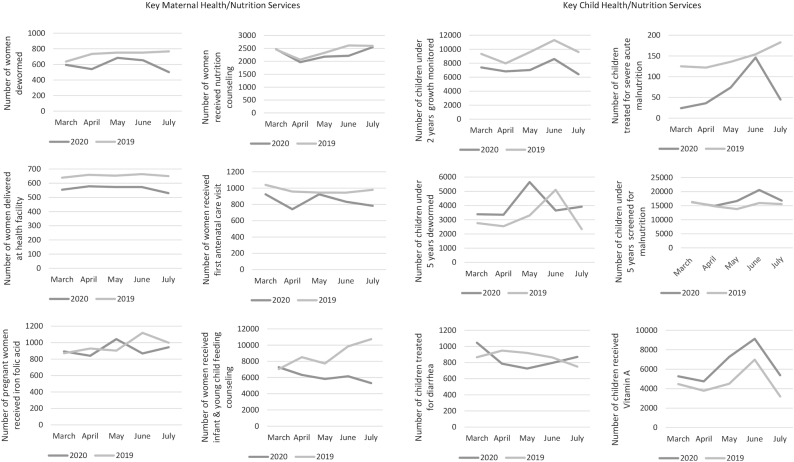
Comparison of trends in key maternal health/nutrition service and child health/nutrition service utilization at health centers during March to July 2019 and 2020.

The trends in selected maternal nutrition and health service utilization were evaluated by comparing data from the same HCs from March to July in 2019 and 2020. As shown in **Figure 1**, rates of women's deworming, nutrition counseling, delivery at a health facility, and receipt of antenatal care, were somewhat lower in 2020 compared with the previous year, while there was a much larger difference in infant and young child feeding counseling services. At the HP level, pregnant women conference, maternal deworming, and complementary food demonstration were the maternal health services that were much lower in 2020. For children under 5 years of age, growth monitoring and treatment for severe acute malnutrition at HCs were lower in 2020 relative to 2019 (Figure 1). While other child services also showed some small difference between years, there is a noticeable difference in provision of Vitamin A supplementation, which was consistently higher in 2020 compared with 2019.

According to respondents, several mitigation strategies were put in place to offset the negative effects on health service delivery and utilization, with a strong emphasis on awareness creation for the community. The awareness creation focused on 2 main messages, stressing community-level prevention techniques for COVID-19, and messaging focused on COVID-19 prevention techniques to use when coming to HCs. In order to expand the cadre of people who could inform communities and households, volunteers were recruited from university students, frontline community workers, religious leaders, and others. At the same time, other strategies to reach households included providing counseling over the phone and health extension workers initiating a home-visit program. This approach is captured in the following:

We are giving door-to-door awareness creation services. On the other hand, we have created awareness to the community and health extension workers about how to resume activities which request gathering of people by fulfilling all necessary prevention mechanisms including social distance in place … We have made telephone counseling service available to mothers for maternal and child health services. (KI from HC in Tigray)

We tried to make health workers to create awareness door-to-door. We tried to give door-to-door service for activities which are possible to provide at the household level. (KI with HEW from Oromia)

## Discussion

Routine health services in general, and maternal and child health and nutrition services in particular, for the most part showed some decline in comparison with the same time period in the previous year in the study areas. Most of the declines in utilization of services occurred in the early period of the pandemic—from March to April 2020—when coordinated efforts and standard guidance were in the early stages, but most services had recovered by the end of July. Initial concern and lack of awareness of the nature of COVID-19 was reported as a significant factor associated with lower utilization of health services. Another major influence that negatively affected health-care services was the prohibition of mass gatherings combined with travel restrictions. The higher rates of Vitamin A supplementation in 2020 may have been due to the transition of supplementation from facility-based to outreach, home-based service.

The mitigation efforts of the government and other agencies did appear to be effective, to a certain extent, in reversing downward trends in service utilization. The awareness creation efforts at the community level improved the health-seeking behavior of households. These activities were also very much in line with the guidance from the FMoH to spread information on basic infection prevention and guide safe care-seeking behavior (10). This, along with other adaptive measures such as virtual counseling and house-to-house visits, helped offset some of the challenges in obtaining or delivering services. Another key lesson learned was the importance of redesigning the provision of services that required mass gatherings by limiting the number of participants and simultaneously increasing the number of events to deliver health-care activities.

While health providers reported that modifications have been successful in reviving services, continued support to ensure that adequate funding and supplies are available for modified services and directly reaching communities and households will be important to sustain ongoing efforts and ensure the health system can continue to meet community needs as the COVID-19 pandemic continues.

### Study Limitations

One of the study limitations was potential quality issues in the health data collected from health facilities. As the data was collected over the phone, it was also verified with copies of the original records to the extent possible, but studies of Ethiopian health facility monitoring data have identified high rates of inaccuracies in records (11–12). While the original study design also included records from 2018, poor quality and incompleteness of the 2018 data made it unusable. The study was thus not able to identify normal annual variation patterns for comparison. Another limitation of the study was that the representatives selected for both the quantitative and the qualitative data collection were likely to have been engaged in the design and implementation of the mitigation strategies and thus likely to have been biased regarding their effectiveness.

## Notes

Author disclosures: The authors report no conflicts of interest.

Abbreviations used: FMoH, Federal Ministry of Health; HC, health center; HEW, health extension worker; HP, health post; KI, key informant; PHCU, Primary Health Care Unit; PPE, personal protective equipment.
